# Prognostic value of lymph node ratio in patients with non-small cell lung cancer: a systematic review and meta-analysis

**DOI:** 10.3389/fonc.2025.1601575

**Published:** 2025-07-01

**Authors:** Junhuang Zheng, Haoyu Zhong, Enxiang Quan, Zhiyan Gao, Songsong Ying

**Affiliations:** ^1^ Institute of Gerontology, Guangzhou Geriatric Hospital, Guangzhou Medical University, Guangzhou, China; ^2^ State Key Laboratory of Respiratory Disease, Guangzhou Geriatric Hospital, Guangzhou Medical University, Guangzhou, China; ^3^ Collaborative Innovation Center for Civil Affairs of Guangzhou, Guangzhou, China; ^4^ Department of Gastroenterology, Guangzhou Geriatric Hospital, Guangzhou Medical University, Guangzhou, China; ^5^ Department of Clinical Medicine, The Nanshan College of Guangzhou Medical University, Guangzhou, China; ^6^ Department of Clinical Medicine, The Third Clinical School of Guangzhou Medical University, Guangzhou, China; ^7^ Department of Clinical Medicine, Clinical College of Integrated Traditional Chinese and Western Medicine, Guangzhou Medical University, Guangzhou, China; ^8^ Department of Morphology Experiment Center, Guangzhou Medical University, Guangzhou, Guangdong, China

**Keywords:** lymph node ratio, lymph node metastasis, lung cancer, non-small cell prognostic predictive efficacy, meta-analysis

## Abstract

**Background and purpose:**

The lymph node ratio (LNR), by indirectly quantifying the dynamic balance between metastatic burden and host immune clearance, may provide more accurate prognostic stratification information. This meta-analysis aims to evaluate the prognostic value of LNR in patients with non-small cell lung cancer (NSCLC).

**Methods:**

A systematic literature search was conducted in PubMed, Embase, Cochrane, and Web of Science databases, with the search date up to January 7, 2025. Studies were strictly selected based on pre-specified inclusion and exclusion criteria. Data were merged and analyzed using Stata 16.0.

**Results:**

This meta-analysis included 11 studies. High LNR was significantly associated with decreased overall survival (OS) (multivariable HR=1.76, 95% CI=1.36-2.27; univariable HR=2.26, 95% CI=1.95-2.63) and increased risk of shorter disease-free survival (DFS) (multivariable HR=1.66, 95% CI=1.48-1.88). Subgroup analysis showed that regardless of whether the LNR cutoff was set at >0.25 (OS-HR=1.62; DFS-HR=1.82) or ≤0.25 (OS-HR=2.02; DFS-HR=1.58), high LNR indicated poor prognosis. Heterogeneity analysis showed high heterogeneity for OS outcomes (I²=91.8%) and low heterogeneity for DFS outcomes (I²=21.5%). After publication bias was corrected by trim-and-fill method, the combined effect size remained stable.

**Conclusion:**

LNR is an independent prognostic factor for survival in NSCLC patients. Future prospective studies are needed to optimize the LNR cutoff values and integrate molecular biomarkers to construct precise prognostic models, which could provide evidence for updating the TNM staging system and personalized treatment.

**Systematic Review Registration:**

https://www.crd.york.ac.uk/PROSPERO/view/CRD420251022895, identifier (CRD420251022895).

## 1 Introduction

Lung cancer, one of the leading malignancies in terms of incidence and mortality worldwide, causes approximately 1.8 million deaths each year, posing a severe threat to human health (1, 2). The two main types of lung cancer are small cell lung cancer (SCLC) and non-small cell lung cancer (NSCLC), with NSCLC accounting for 85% of lung cancer cases (3). Epidemiological data shows that the mortality rate from lung cancer is particularly prominent in specific populations, with the number of deaths from lung cancer exceeding those from breast cancer, prostate cancer, colorectal cancer, and leukemia in men over 40 years old and women over 60 years old, highlighting its severe public health burden (4).

Lymph node metastasis (N) is a core indicator in the pathological staging of NSCLC, as defined by the TNM staging system. The extent of lymph node metastasis (e.g., N1/N2/N3) directly determines disease staging, treatment strategies, and prognosis (5). However, the traditional N staging, which relies on the number of positive lymph nodes, has increasingly shown its limitations. Firstly, differences in the number of lymph nodes sampled or the methods of examination may lead to staging bias. Secondly, the biological heterogeneity of lymph node metastasis (such as micrometastasis, skip metastasis) and the dynamic interaction between the tumor and the host immune microenvironment make it difficult for traditional N staging to fully reflect disease invasiveness and metastatic burden (6). For example, if patient A undergoes the dissection of 10 lymph nodes (2 positive), and patient B undergoes the dissection of 30 lymph nodes (2 positive), the traditional N staging would both classify them as N1, but their actual metastatic burden may differ. Therefore, updating the current N classification and developing a more accurate N classification system is of great significance (7).

In recent years, the lymph node ratio (LNR), defined as the ratio of the number of positive lymph nodes to the total number of dissected lymph nodes, has gradually become a potential prognostic surrogate marker (8).The advantage of LNR lies in integrating both the absolute number of metastatic lymph nodes and the extent of lymph node dissection, quantifying the balance between local tumor metastasis burden and the host immune clearance capacity (9).LNR plays an important role in predicting patient prognosis and has been validated in diseases such as oral cancer (10), colon cancer (11) and breast cancer (12) Studies on NSCLC also suggest that an elevated LNR is significantly associated with shorter overall survival (OS) and disease-free survival (DFS), and it may even optimize adjuvant treatment stratification for stage II-III patients (13–15).However, existing evidence is highly heterogeneous, and there is no unified standard for the critical value of LNR. Additionally, most studies are retrospective, which are susceptible to selection bias and confounding factors, and the clinical applicability of LNR still requires support from high-quality evidence. Therefore, this study aims to assess the prognostic predictive efficacy of LNR for NSCLC patients through a systematic review and meta-analysis, explore the clinical applicability of LNR, and its potential contribution to precision medicine, providing evidence-based guidance for future clinical research design.

## 2 Materials and methods

### 2.1 Search strategy

Pubmed, Embase, Cochrane, and Web of Science databases were searched from their inception to January 7, 2025. The main keywords included “Carcinoma, Non-Small-Cell Lung” and “Lymph Node Ratio,” with searches conducted using a combination of subject terms and free words. The detailed search strategy is outlined in **Supplementary Material**.

### 2.2 Inclusion and exclusion criteria

Studies were included if they met all of the following criteria (1): Population: Patients diagnosed with non-small cell lung cancer (NSCLC) according to clinical diagnostic criteria who had undergone surgical resection and lymph node dissection (2); Intervention and Comparison: Studies that clearly reported the lymph node ratio (LNR), defined as the ratio of positive lymph nodes to the total number of dissected lymph nodes, and provided comparisons between high and low LNR groups; (3) Outcomes: Reported at least one survival outcome, such as overall survival (OS) or disease-free survival (DFS), in relation to LNR levels; (4) Study design: Cohort studies; (5) Time: Follow-up duration was specified in each included study.

Studies meeting any of the following criteria were excluded:1) Duplicate studies using the same population or overlapping databases; 2) Meta-analyses, systematic reviews, literature reviews, letters, replies, conference abstracts, case reports, guidelines, or consensus statements; 3) Animal or *in vitro* studies.

### 2.3 Literature screening

The retrieved studies were imported into Endnote X9, and duplicate studies were excluded automatically by the software and manually by researchers. A preliminary screening was then conducted by reading the titles and abstracts, followed by downloading the studies that met the initial screening criteria. Full texts were reviewed to confirm eligibility, and only the original studies meeting the criteria for this meta-analysis were included. The literature screening was conducted independently by two researchers (Junhuang Zheng and Zhiyan Gao), and any disagreements were cross-checked. In case of disputes, researcher (Songsong Ying) helped resolve them.

### 2.4 Data extraction

After screening, a specialized Excel data extraction form was designed for this study, and information from the included articles was summarized. The information extracted included:

1. General Information: First author, publication year, country, study type, age, and gender of the study population.
2. Study Characteristics: Intervention measures, exposure levels, analysis methods, risk ratios for outcome measures, and 95% confidence intervals.

For studies with incomplete data, the corresponding authors were contacted. Two reviewers (Junhuang Zheng and Zhiyan Gao) independently extracted data from the selected studies, and any discrepancies were resolved by a third reviewer (HaoyuZhong).

### 2.5 Quality assessment

Two reviewers (HaoyuZhong and Enxiang Quan) independently evaluated the methodological quality of each included cohort study using the Newcastle-Ottawa Scale (NOS), which has three main modules and eight items (16). The evaluation covered three aspects:Selection of the study population (0–4 points), Comparability between groups (0–2 points), Outcome measurement (0–3 points). A total score of 6 or more was considered a high-quality study. Any discrepancies were resolved through discussion or, if necessary, by arbitration from a third reviewer (Songsong Ying).

### 2.6 Statistical analysis

Meta-analysis was performed using Stata 16.0. The hazard ratios (HR) and 95% confidence intervals (95% CI) for the prognostic indicators of LNR levels were directly extracted from the included studies, or univariate data from some studies were estimated using the methods described by Parmar et al.人 (17) and Tierney et al. (18), along with the Engauge 11.3 software and an Excel table for HR and 95% CI calculation. If a study reported multiple estimates, we selected the multivariable analysis results adjusted for confounders. I^2^ was used to assess heterogeneity between studies. A fixed-effect model was applied for I^2^ < 50% (low heterogeneity), and a random-effects model for I^2^ ≥ 50% (high heterogeneity). Subgroup analysis was conducted to explore the sources of heterogeneity, and sensitivity analysis was performed to examine the stability of the study results. Sensitivity analysis involved removing one study at a time to assess its impact on the overall outcome. Begg’s and Egger’s tests were used to assess publication bias. If bias was detected, the trim-and-fill method was used for correction. All p-values were two-tailed, and statistical significance was set at p < 0.05.

## 3 Results

### 3.1 Search results

**Figure 1:**
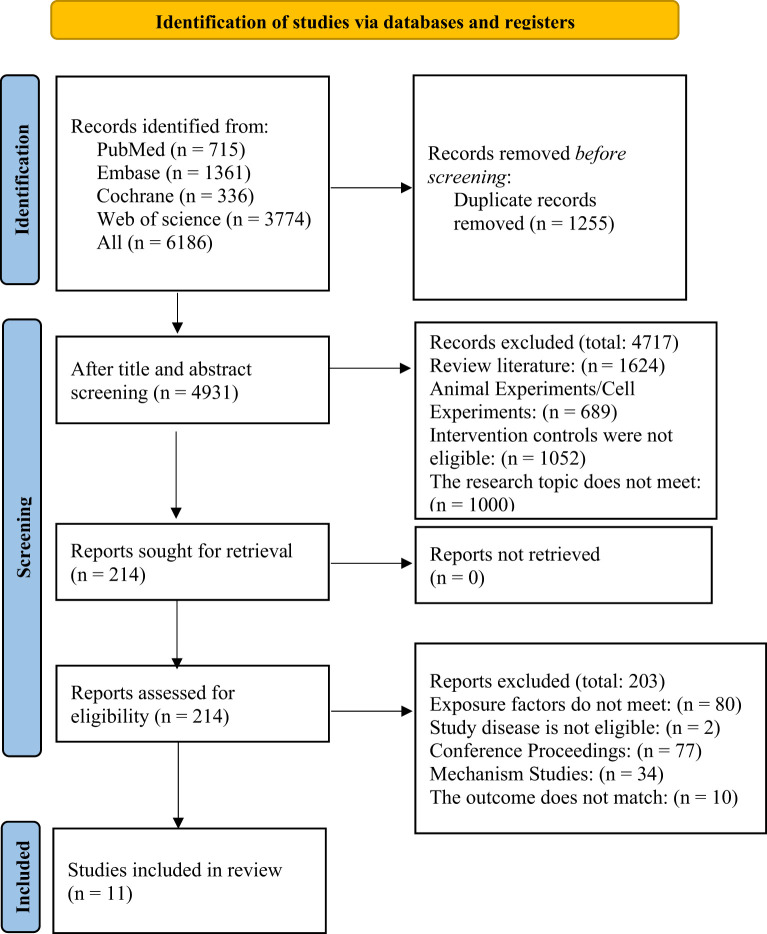
Literature selection process.

A total of 6,186 records were retrieved from four databases. After automatically and manually removing 1,255 duplicates, 4,931 records remained. A preliminary screening of titles and abstracts led to the exclusion of 4,717 studies. The remaining 214 articles were subjected to full-text review. Ultimately, 11 studies (19–29) met all inclusion criteria. The reasons for exclusion and the detailed search flow are shown in **Figure 1**.

### 3.2 Characteristics of included studies

**Table 1** summarizes the basic characteristics of the included studies. A total of 11 studies were included, involving 18,759 participants, with 10,835 men and 7,924 women. Seven studies were from Asia (19, 22–25, 27, 29), two were from Europe (20, 26), and two were from North America (21, 28).The publication years ranged from 2011 to 2024. The LNR cutoff values varied between 0.05 and 0.58. The quality assessment results based on the NOS scale are detailed in **Table 2**, with all studies scoring above 6 points, indicating they were of high quality.

**Table 1:** Baseline table of the literature was included.

No.	First Author	Year	Country	Type	Treatment	Number of cases	Gender (M/F)	Age	Stage [n (%)]	Outcomes
1	He, C	2024 (22)	China	Retrospective study	Radical resection	5249	2735/2514	<60: 1370 ≥60: 3879	T1: 1314 (25.03%) T2: 2641 (50.31%) T3: 984 (18.75%) T4: 310 (5.91%)	OS
2	Wu,C.Y	2015 (29)	Taiwan,China	Retrospective study	Radical resection	422	236/186	62.58 ± 11.09	1a:99 (22.4%) 1b:170 (38.5%) 2a:69 (15.6%) 2b:28 (6.3%) 3a:74 (16.7%) No residual tumor:1 (0.2%) Premalignant lesion:1 (0.2%)	OS
3	Li,Y	2011 (24)	China	Retrospective study	Radical resection	301	198/103	<60: 157 ≥60: 144	T1: 88 (29.2%) T2: 178 (59.2%) T3: 35 (11.6%)	OS, DFS
4	Chen,Z.Y	2024 (19)	China	Retrospective study	Radical resection and subsequent adjuvant chemotherapy	1690	815/875	<66: 824 ≥66: 866	T1: 563 (33.31%) T2: 1127 (66.69%)	OS
5	Hsieh, C. P	2015 (23)	Taiwan	Retrospective study	Radical resection and postoperative adjuvant therapy	108	56/52	60.2± 11.5	T0: 1 (0.9%) T1a: 10 (9.3%) T1b: 13 (12.0%) T2a: 60 (55.7%) T2b: 18 (16.7%) T3: 6 (5.6%)	OS, DFS
6	Renaud, S	2015 (26)	France	Retrospective study	Radical resection and neoadjuvant treatment	152	121/31	NA	T0: 5 (3.3%) T1: 39 (25.7%) T2: 78 (51.3%) T3: 29 (19.1%) T4: 1 (0.7%)	OS
7	Li, Z. M	2013 (25)	China	Retrospective study	Radical resection	206	166/40	64	NA	OS, DFS
8	Chiappetta, M	2019 (20)	Italy	Retrospective study	Anatomical lung resection and hilome diastinal lymphadenectomy	4858	3405/1453	66.7± 17.7	T1: 1548 (32.3%) T2: 2221 (47.1%) T3: 654 (13.2%) T4: 283 (5.7%) Missing data: 152 (0.9%)	OS, DFS
9	Deng, W	2018 (21)	United States	Retrospective study	Lobectomy or pneumonectomy	5289	2793/2496	<50: 243 50-59: 945 50-69:1886 70-79:1710 ≥80: 505	T1: 1467 (27.7%) T2: 2687 (50.8%) T3: 905 (17.1%) T4: 210 (4.0%)	OS
10	Tamura, M	2016 (27)	Japan	Retrospective study	Surgically complete resection with a systematic lymphadenectomy	182	127/55	64.6	T1: 75 (41.2%) T2: 87 (47.8%) T3: 18 (9.9%) T4: 2 (1.1%)	OS
11	Taylor, M. D	2013 (28)	United States	Retrospective study	R0 resection	302	183/119	NA	IIA: 142 (47.0%) IIB: 51 (16.9%) IIIA: 106 (35.1%) IIIB: 3 (1.0%)	OS

**Table 2:** NOS quality evaluation.

Study	Queue selection				Comparability	Outcome			
	Representativeness of the exposure cohort	Representativeness of the unexposed cohort	Determination of exposure	None of the study subjects had developed the disease under study at the start of the study	Comparability of exposed and unexposed cohorts	Methods for determining results	Whether the follow-up was long enough	Completeness of follow-up	Overall score
He, C 2024 (22)	1	1	1	1	2	1	0	1	8
Wu, C.Y 2015 (29)	1	1	1	1	2	1	1	0	8
Li, Y 2011 (24)	1	1	1	1	2	1	1	0	8
Chen, Z.Y 2024 (19)	1	1	1	1	2	1	1	0	8
Hsieh, C. P 2015 (23)	1	1	1	1	2	1	1	1	9
Renaud, S 2015 (26)	1	1	1	1	2	1	0	0	7
Li, Z. M 2013 (25)	1	1	1	1	2	1	1	1	9
Chiappetta, M 2019 (20)	1	1	1	1	2	1	1	1	9
Deng, W 2018 (21)	1	1	1	1	1	1	1	0	7
Tamura, M 2016 (27)	1	1	1	1	1	1	1	0	7
Taylor, M. D 2013 (28)	1	1	1	1	1	1	1	1	8

### 3.3 Overall and subgroup analysis of overall survival

**Figure 2:**
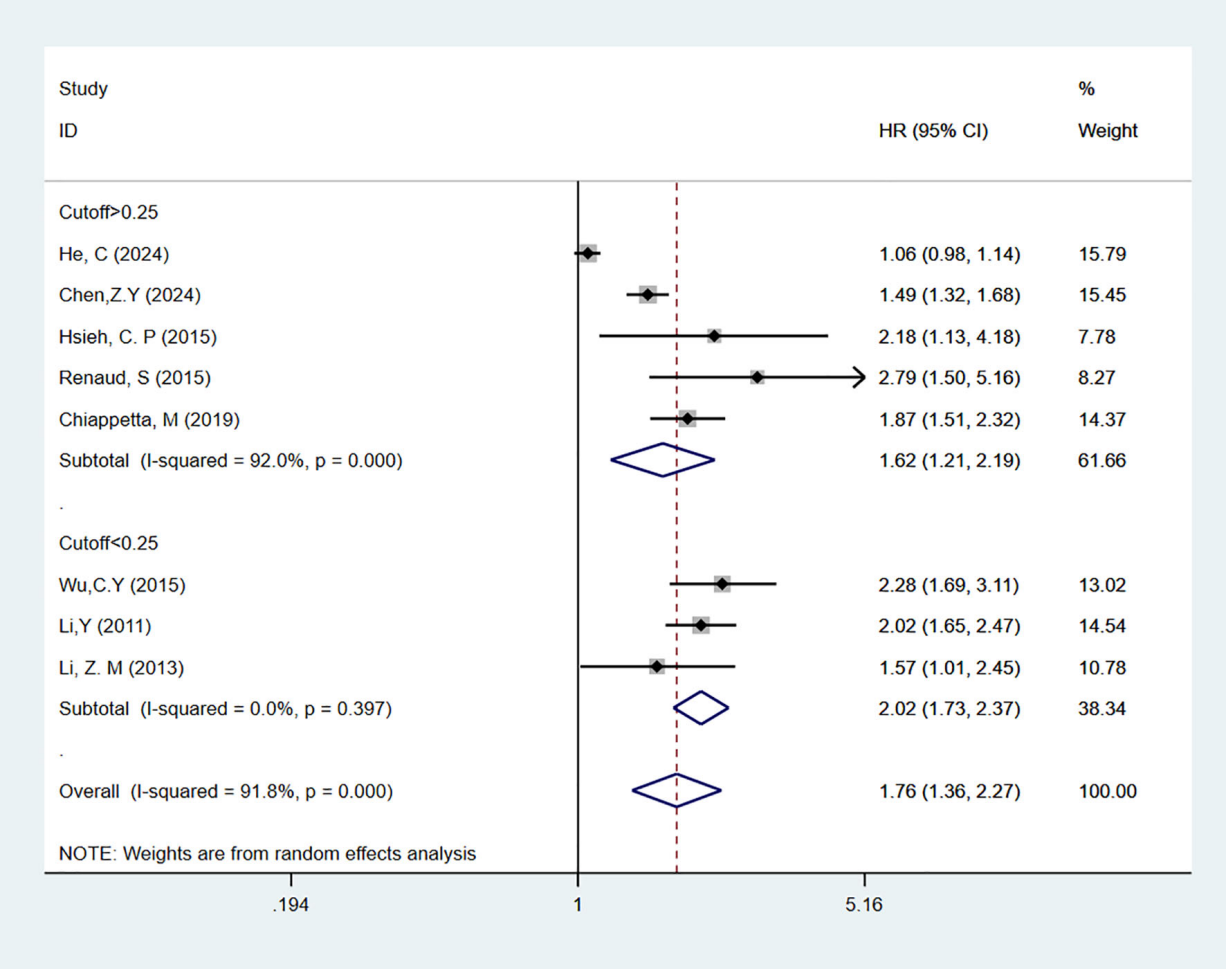
Forest plot showing the correlation between high LNR and OS in multivariable analysis.

**Figure 3:**
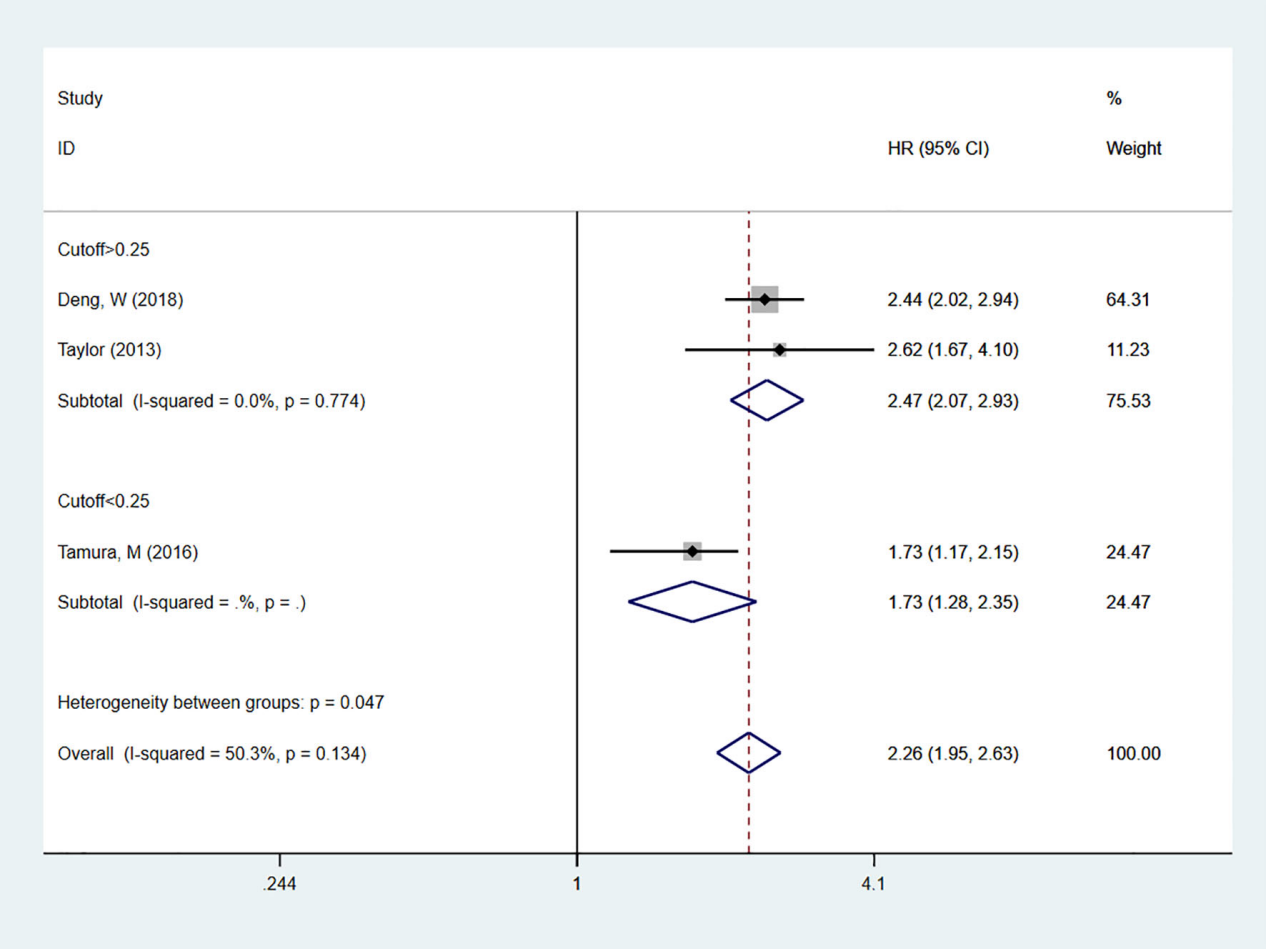
Forest plot showing the correlation between high LNR and OS in univariate analysis. The group with cut-off < 0.25 includes only one study, so only the effect estimate of that study is shown individually. The diamond in the figure represents the pooled effect estimate of all included studies, reflecting the overall univariate analysis results.

A total of 11 studies (19–29) reported on LNR as a predictor for OS in patients. In multivariable analysis, with high heterogeneity [I^2^ = 91.8%], a random-effects model was used. The results indicated that high LNR levels were associated with poor OS in NSCLC patients [HR = 1.76, 95% CI = 1.36-2.27]. Subgroup analyses with cutoff values > 0.25 [HR = 1.62, 95% CI = 1.21-2.19] or < 0.25 [HR = 2.02, 95% CI = 1.73-2.37] also showed that high LNR is a predictor of poor OS in NSCLC patients (**Figure 2**). In univariate analysis [I^2^ = 50.3%], the results showed that high LNR was still associated with poor OS in NSCLC patients [HR = 2.26, 95% CI = 1.95-2.63]. Subgroup analyses with cutoff values > 0.25 [HR = 2.47, 95% CI = 2.07-2.93] or < 0.25 [HR = 1.73, 95% CI = 1.28-2.35] confirmed that high LNR was a predictor of poor OS (**Figure 3**).

### 3.4 Overall and subgroup analysis of disease-free survival

**Figure 4:**
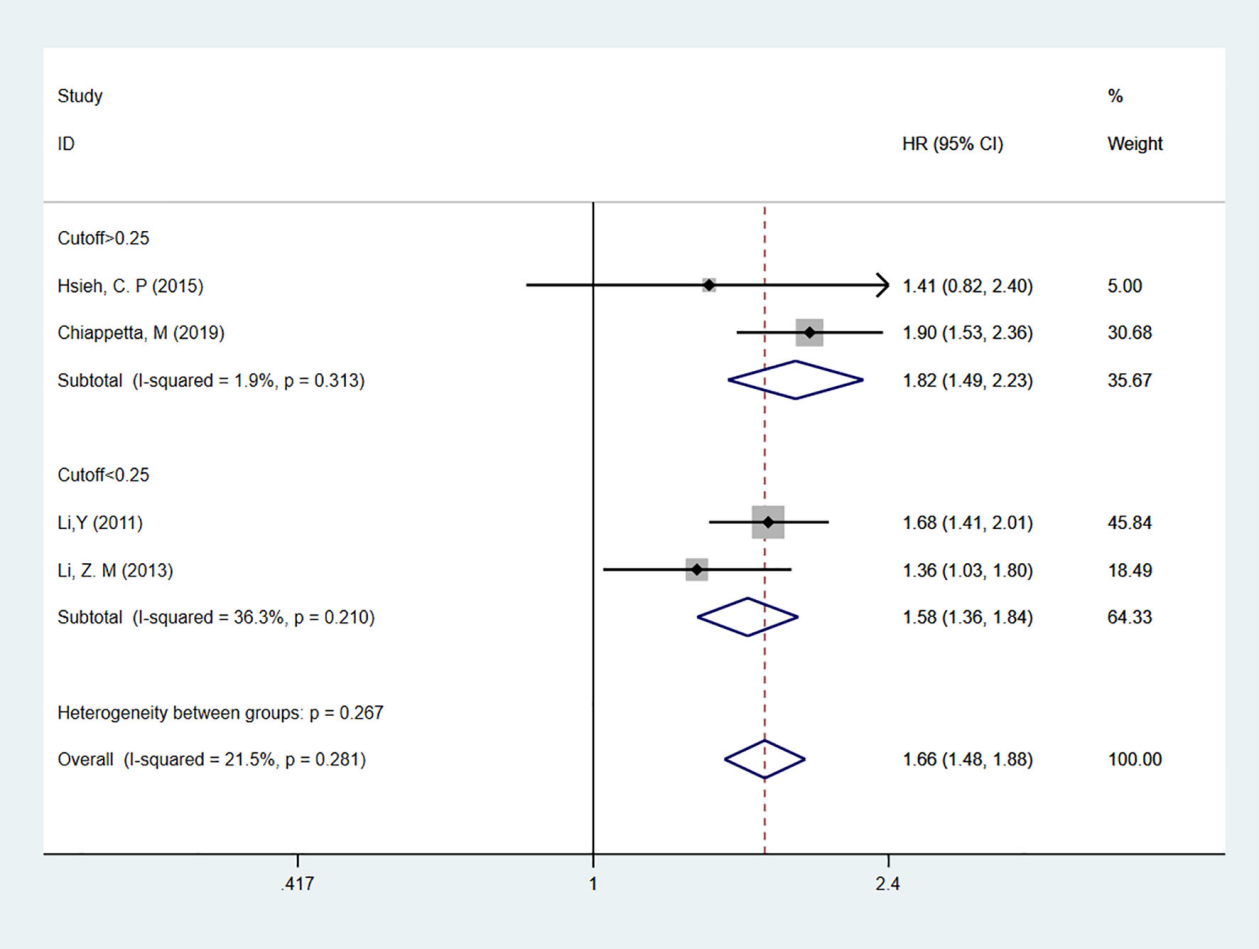
Forest plot showing the correlation between LNR levels and DFS (multivariable analysis).

Four studies (20, 23–25) reported on LNR as a predictor for DFS in patients, all using multivariable analysis. The heterogeneity test results showed I^2^ = 21.5%, and a fixed-effect model was used for analysis. The overall result indicated that high LNR levels predicted shorter DFS in patients [HR = 1.66, 95% CI = 1.48-1.88]. Subgroup analyses based on cutoff values showed that for cutoff > 0.25 [HR = 1.82, 95% CI = 1.49-2.23] or cutoff < 0.25 [HR = 1.58, 95% CI = 1.36-1.84], high LNR remained a predictor of poor DFS in NSCLC patients (**Figure 4**).

### 3.5 Sensitivity analysis and publication bias

**Figure 5:**
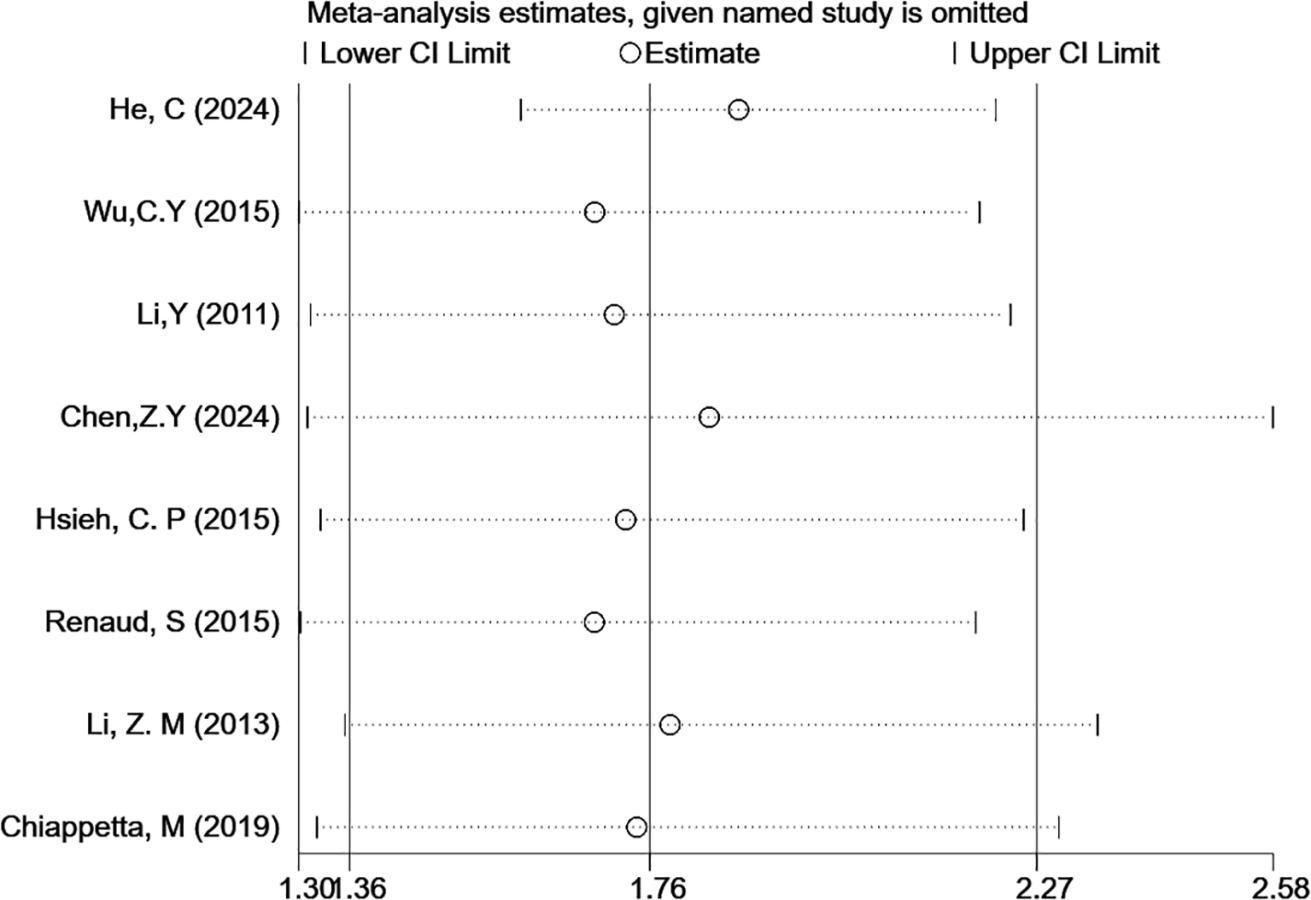
Sensitivity analysis of the correlation between LNR levels and OS (multivariable analysis).

Sensitivity analysis and publication bias tests were conducted for outcomes with at least five included studies. Each study was removed one at a time for sensitivity analysis to assess the stability of HRs for OS. The analysis results suggested that the sensitivity was low, and the results were stable (**Figure 5**). Egger’s test for publication bias indicated that P(OS) = 0.02, which is less than 0.05, suggesting the presence of publication bias. Therefore, the trim-and-fill method was used to correct for publication bias, as detailed in **Supplementary Material 2**.

## 4 Discussion

Through systematic review and meta-analysis, this study suggests that LNR is an important independent prognostic indicator for survival in patients with NSCLC. The results show that high LNR levels are significantly associated with reduced OS and DFS, a finding that is highly consistent with several previous studies (20, 30, 31). Notably, a large-scale analysis based on the SEER database revealed that the LNR-based prognostic stratification achieved a C-index of 0.71, significantly outperforming the discriminative ability of the traditional N staging, suggesting its potential advantage in clinical prognostic evaluation (32). Furthermore, subgroup analysis of LNR showed that different cutoff values could distinguish patient prognosis. Although there is no unified standard for defining the cutoff value, the overall trend remains consistent: the higher the LNR, the poorer the prognosis.

In the current 8th edition of the TNM staging system by the International Association for the Study of Lung Cancer (IASLC) (33), the N category is still based on the anatomical location of metastatic lymph nodes (34). However, substantial clinical evidence indicates that even within the same N staging subgroup, there is significant heterogeneity in clinical outcomes, particularly in N1 and N2 patient groups where overlapping survival outcomes are observed (35, 36). Compared to traditional N staging, LNR effectively avoids the classification bias caused by differences in lymph node dissection range, as it combines the number of positive lymph nodes with the total number of dissected lymph nodes (15, 20). This study’s multivariable analysis further confirms that LNR remains significantly associated with both OS and DFS, independent of other clinical and pathological factors, highlighting its robustness as a prognostic biomarker.

From a biological mechanism perspective, LNR may affect prognosis through dual pathways: on one hand, a higher LNR directly reflects a larger tumor burden and more active lymphatic metastatic potential (37); on the other hand, it may be associated with the formation of an immunosuppressive microenvironment (38). These findings provide potential pathophysiological explanations for the prognostic predictive value of LNR.

From a methodological perspective, this study shows that the heterogeneity of LNR in predicting OS for NSCLC patients is high (I² = 91.8%), which may be related to factors such as the patient populations, treatment regimens, and LNR cutoff values in the included studies (39). However, subgroup analyses revealed that different cutoff groups (cut-off = 0.25) maintained the stability of prognostic discrimination (all p < 0.05). It is worth noting that the heterogeneity in the DFS analysis was relatively low (I² = 21.5%), likely due to the higher homogeneity of treatment protocols in the included studies. After adjusting for publication bias using the trim-and-fill method, the combined effect size remained statistically significant, confirming the reliability of the study’s conclusions.

The strengths of this study lie in its strict adherence to the methodology of systematic reviews and meta-analysis, with all included studies being high-quality cohort studies that employed multivariable analysis to control for confounding factors, ensuring the robustness of the results. Additionally, subgroup and sensitivity analyses were conducted to explore sources of heterogeneity, enhancing the reliability of the study. However, there are some limitations. First, the optimal cutoff value for LNR has not been standardized, which may lead to heterogeneity between studies (40). Second, most of the included studies were retrospective, which could introduce selection bias and information bias. Third, although we attempted to contact the original authors for missing data, some studies still lacked complete survival analysis data, which may affect the precision of the meta-analysis. Moreover, the multivariable models varied across studies, and some did not adjust for important confounders such as resection margins or the extent of lymph node dissection, which may impact the comparability and internal validity of the results. The prognostic significance of LNR may also vary depending on the extent of lymph node dissection. However, the available data did not allow stratification by total lymph node count, which should be investigated in future studies. In addition, molecular biomarkers and other potential prognostic factors were not included, which could influence the independent predictive value of LNR. Due to limited data availability, this study was also unable to assess the relationship between LNR and specific lymph node stations (N1, N2, N3). Further investigation using individual patient data is warranted to explore the prognostic interaction between these variables more comprehensively. Lastly, most of the included studies were conducted in Asian populations, which are known to have distinct molecular profiles and treatment responses compared to Western populations. This may limit the generalizability of our findings, and future multinational studies are needed to validate these results.

Despite these limitations, the prognostic value of LNR may aid clinicians in stratifying patients for adjuvant therapy, particularly in borderline-stage cases. For example, patients with a high LNR may benefit from more intensive postoperative treatment or closer follow-up. Incorporating LNR into clinical decision-making tools could enhance personalized management strategies in resected NSCLC.

## 5 Conclusion

This study confirms that LNR is an independent predictor of OS and DFS in patients with NSCLC, with higher LNR levels indicating poorer survival prognosis. Future research should focus on determining the optimal cutoff value for LNR and combining it with other biomarkers to optimize staging and treatment strategies for NSCLC. Moreover, large-scale prospective studies and randomized controlled trials are necessary to validate the clinical decision-making value of LNR and provide more precise evidence for personalized treatment.

## Data Availability

The original contributions presented in the study are included in the article/**Supplementary Material**. Further inquiries can be directed to the corresponding authors.
