# Supplementary figures and images for: Prognostic value of lymph node ratio in patients with non-small cell lung cancer: a systematic review and meta-analysis
Source: Front Oncol. 2025 Jul 1;15:1601575. doi: 10.3389/fonc.2025.1601575 (PMC12259456; doi:10.3389/fonc.2025.1601575)

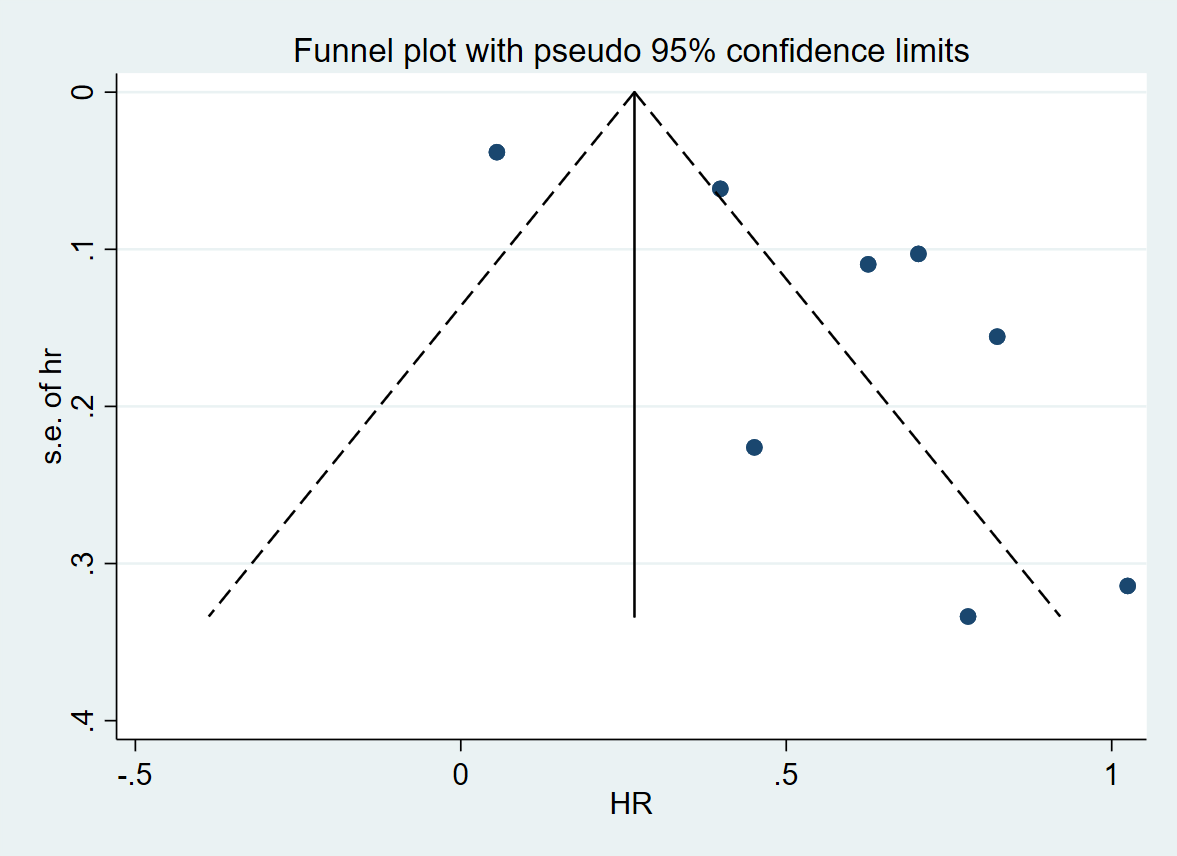

Supplement: Supplementary file 2 [file DataSheet2.zip › Supplementary Material 2 published tests of bias and trimming/LNR-OS multivariate funnel plot.tif]
